# Reappraisal generation after acquired brain damage: The role of laterality and cognitive control

**DOI:** 10.3389/fpsyg.2014.00242

**Published:** 2014-03-21

**Authors:** Christian E. Salas, James J. Gross, Oliver H. Turnbull

**Affiliations:** ^1^Centre for Cognitive Neuroscience, School of Psychology, Bangor UniversityBangor, UK; ^2^Unidad de Psicoterapia Dinámica, Instituto Psiquiátrico J. H. BarakSantiago, Chile; ^3^Department of Psychology, Stanford UniversityStanford, CA, USA

**Keywords:** brain injury, emotion regulation, reappraisal, cognitive control, emotion, inhibition, verbal fluency

## Abstract

In the past decade, there has been growing interest in the neuroanatomical and neuropsychological bases of reappraisal. Findings suggest that reappraisal activates a set of areas in the left hemisphere (LH), which are commonly associated with language abilities and verbally mediated cognitive control. The main goal of this study was to investigate whether individuals with focal damage to the LH (*n* = 8) were more markedly impaired on a reappraisal generation task than individuals with right hemisphere lesions (RH, *n* = 8), and healthy controls (HC, *n* = 14). The reappraisal generation task consisted of a set of ten pictures from the IAPS, depicting negative events of different sorts. Participants were asked to quickly generate as many positive reinterpretations as possible for each picture. Two scores were derived from this task, namely *difficulty* and *productivity*. A second goal of this study was to explore which cognitive control processes were associated with performance on the reappraisal task. For this purpose, participants were assessed on several measures of cognitive control. Findings indicated that reappraisal difficulty – defined as the time taken to generate a first reappraisal – did not differ between LH and RH groups. However, differences were found between patients with brain injury (LH + RH) and HC, suggesting that brain damage in either hemisphere influences reappraisal difficulty. No differences in reappraisal productivity were found across groups, suggesting that neurological groups and HC are equally productive when time constraints are not considered. Finally, only two cognitive control processes inhibition and verbal fluency- were inversely associated with reappraisal difficulty. Implications for the neuroanatomical and neuropsychological bases of reappraisal generation are discussed, and implications for neuro-rehabilitation are considered.

## INTRODUCTION

Emotion dysregulation has been long recognized as a common impairment after focal and diffuse brain damage, compromising emotional adjustment, and social functioning (Tate, 1999; Bechara, 2004; Obonsawin et al., 2007; Abreu et al., 2009; McDonald et al., 2010). However, little is known about the mechanisms that underlie such impairment. The process model of emotion regulation (ER; Gross, 1998, 2014; Gross and Thompson, 2007) suggests the existence of a set of five mechanisms, called strategies, that people commonly use to modulate how they feel. Recently, it has been proposed that these strategies may be selectively impaired in groups of patients with damage to discrete brain areas (Beer and Lombardo, 2007).

Reappraisal is an ER strategy of special interest to understand emotion dysregulation after brain injury. It refers to the capacity to modify the meaning of a situation in order to manipulate its emotional impact (Gross, 1998; Gross and Thompson, 2007), this by re-interpreting the event in less negative, or more positive, terms (Ochsner and Gross, 2007; Mcrae et al., 2012a). This capacity to manipulate emotion through the use of reappraisal has been referred to as reappraisal *ability*. Most behavioral and neuroimaging studies on reappraisal have focused on this global ability, without directly considering the assessment of people’s capacity to produce new interpretations of negative events (reappraisal *generation*). Such emphasis on *ability* is sensible, considering that people with healthy brains probably have an intact capacity to *generate* reappraisals. However, individuals with brain damage, particularly to the prefrontal cortex, are often impaired in the generation and manipulation of thoughts (Goldstein, 1936; Goldstein and Scherer, 1941; Luria, 1966; Gomez Beldarrain et al., 2005). If reappraisal relies on the flexible use of thinking (Ochsner and Gross, 2004), then it is likely that people with brain injury may struggle using such strategy to modulate how they feel.

From a neuropsychological point of view, it has been suggested that reappraisal depends on capacities like *inhibition *(to inhibit the current negative appraisal and generate a new one) and *language* (to generate a narrative or history to tell oneself; Mcrae et al., 2012b). These abilities are frequently compromised after lesions to the left hemisphere (LH; Luria, 1959, 1966; Mecklinger et al., 1999; Alexander et al., 2007; Morin, 2009; Robinson et al., 2010; Geva et al., 2011; Coelho et al., 2012).

Neuroimaging studies on reappraisal appear to support the relevance of the LH. It has been reported that reappraisal is closely associated to the activation of cognitive control and language areas, particularly in the left PFC (Ochsner et al., 2002, 2004; Ochsner and Gross, 2005; Green and Malhi, 2006; Goldin et al., 2008; Kalisch, 2009; Kim et al., 2011; Vanderhasselt et al., 2012). To our knowledge there is only one case study that has explored how damage to the LH (a fronto-parietal lesion), and impairment to cognitive control and language areas, is related to reappraisal generation difficulties (Salas et al., 2013). However, no group study has tested whether unilateral lesions to the LH have a larger impact on reappraisal generation compared to right hemisphere (RH) lesions.

Finally, researchers have begun to unpack the cognitive control processes associated with reappraisal in non-brain injured subjects. It has been suggested that working memory, response inhibition, abstract reasoning, and verbal fluency may be necessary to reappraise (Mcrae et al., 2012b). Nevertheless, evidence has only supported a relationship between working memory and reappraisal ability (Schmeichel et al., 2008; Mcrae et al., 2012b). The study of people with brain damage may contribute to this line of research by exploring whether impairment in a specific neuropsychological capacity (e.g., verbal fluency) is related to reappraisal generation ability.

This is the first study to experimentally test whether lesion laterality has an effect on reappraisal generation. In addition, this study is also the first to explore the relationship between cognitive control impairment and the capacity to generate reappraisals. For this purpose, participants with unilateral lesions to the left and RH, and matched healthy controls, were tested on a reappraisal generation task. In order to obtain a profile of neuropsychological impairment, a set of cognitive tasks was administered. Additionally, measures of emotional symptomatology and ER were collected.

Based on the available literature, this article explored two hypothesis: (1) that participants with LH lesions will present a more marked impairment generating reappraisal compared to subjects with RH lesions and controls (*LH Reappraisal Hypothesis*); (2) that cognitive control abilities, such as, response inhibition, working memory, verbal fluency, and abstraction will be negatively associated to reappraisal difficulty and positively associated to reappraisal productivity (see *Cognitive Control Hypothesis*).

## MATERIALS AND METHODS

### PARTICIPANTS

Participants with unilateral lesions and healthy controls were referred by neurologists from the School of Psychology at Bangor University, this after ethical approval was obtained from the same institution and the Betsi Cadwaladr University Health Board (Wales, UK). The main inclusion criterion for the neurological group was to have a unilateral focal brain lesion. Several exclusion criteria were considered, such as time since injury (no less than 6 months) and language ability (no severe language impairment).

The overall sample involved a total of 30 participants, including individuals with left hemisphere damage (*LH*, *n* = 8, female = 4, male = 4), right hemisphere damage (*RH*, *n* = 8, female = 5, male = 3), and healthy controls (*HC*, *n* = 14, female = 9, male = 5). No differences were found between the three groups in terms of age [LH: *M* = 62.83, *SD* = 9.98; RH: *M* = 57.43, *SD* = 13.34; HC: *M* = 62.86, *SD* = 4.28; *F*(2,28) = 0.31, *p* = 0.73] or education [LH: *M* = 12.83, *SD* = 1.3; RH: *M* = 14.14, *SD* = 1.3; HC: *M* = 14, *SD* = 1.8; *F*(2,28) = 0.16, *p* = 0.85]. In addition, both neurological groups showed no significant differences in terms of months since the injury [LH: *M* = 69.16, *SD* = 49.19; RH: *M* = 63.22, *SD* = 44.35; *t*(14), *p* = 0.47]. For details on lesion location and etiology see **Table 1**.

**Table 1:** Clinical details of left hemisphere and right hemisphere groups.

Lesion latrality	Age/Sex	Etiology	Months since onset	Location
Left hemisphere	52 F	MCA stroke	104	Left prefrontal
	76 F	MCA stroke	25	Left prefrontal, insula
	64 F	MCA stroke	129	Left temporo-parietal
	76 M	MCA stroke	65	Left temporo-parietal
	49 M	Herpes encephalitis	24	Left hippocampus, amygdala, insula
	59 M	MCA stroke	48	Left temporo-parietal
	57 M	MCA stroke	126	Left temporo-parietal
	72 M	MCA stroke	72	Left fronto-parietal
Right hemisphere	57 F	MCA stroke	84	Right prefrontal, insula
	50 M	MCA and ACA stroke	20	Right prefrontal
	45 M	ACoA SAH	70	Right prefrontal
	74 M	MCA Stroke	20	Right ventro-lateral prefrontal cortex, basal ganglia
	46 F	MCA stroke	114	Right prefrontal, insula
	66 F	MCA stroke	120	Right parietal
	78 F	MCA stroke	13	Right prefrontal insula
	68 F	MCA stroke	24	Right prefrontal and parietal

*ACoA, anterior communicating artery aneurism; SAH, subarachnoid haemorrhage; MCA, middle cerebral artery; ACA, anterior cerebral artery.*

### PROCEDURE

Healthy controls and participants with brain injury were tested at Bangor University. In cases where participants with acquired brain injury had mobility problems or could not travel, they were seen at home. Eligible participants were seen twice. Assessment across two sessions was useful to avoid the impact of fatigue on the neurological group. During the first session the general goal of the research was explained and consent was obtained. Measures of overall cognition were also collected. In the second session the reappraisal task was carried and measures of executive function were obtained. Finally participants were debriefed. The complete assessment process was carried by a clinical neuropsychologist. Only the reappraisal task was administered using a computer.

#### Instruments

***Overall cognitive assessment.*** Several tasks were used in order to obtain a general profile of cognitive functioning. The Mini Mental State Examination (Rovner and Folstein, 1987) was used as a basic screening for cognitive impairment. The capacity to sustain attention and divide attentional resources between tasks was assessed using the Telephone Search (*TEA*, Robertson et al., 1994). The ability to comprehend verbal instructions was measured using the Token Test (De Renzi and Faglioni, 1978). A logic memory task (Wechsler, 1987) was used to assess participants’ capacity to register and recall new verbal information. Similarly, the Rey-Osterrieth Figure (Stern et al., 1994) was used to assess visual memory as well as visuo-spatial abilities. Finally, the Frontal Assessment Battery (Dubois et al., 2000) was employed as a screening for executive abilities.

***Cognitive control assessment***. A set of neuropsychological tasks was used to obtain a profile of several cognitive control abilities:

(a) *Working memory *was measured using digits forward and backward (Wechsler, 1981). In the digit forward task participants listened to a series of single-digit numbers and repeat them in the same order. The number of digits in each series increased from 2 to 9. The digit backward task had the same structure, but the participant repeated the numbers in reverse order.
(b) *Abstraction* ability was assessed using the s*imilarities *task (Wechsler, 1981), where participants are instructed to think in which way two items are alike (e.g., boat/car: “means of transport”).
(c) *Verbal fluency* was measured using a subtask from the *D-KEFS* (Delis et al., 2001). Here, participants had to initiate a verbal response and retrieve specific information in accordance with specific rules (e.g., in 1 min, say as many words as you can think of that start with the letter “a”).
(d) *Inhibition* ability was assessed using three measures: a confliciting instructions task (Stuss and Benson, 1986), an inhibitory control task (Drewe, 1975) and an environmental autonomy task (Lhermitte et al., 1986). In the conflicting instructions task participants must provide an opposite response to the examiner’s alternating signals, following verbal command and withholding automatic responses based on visual input (e.g., tapping once when the examiner taps twice). In the inhibitory control task subjects must inhibit a response that was previously given to the same stimulus (e.g., no tapping when the examiner taps twice). In the environmental autonomy task individuals must inhibit the activation of patterns of behavior triggered by sensory stimuli (e.g., the participants places his hands on top of the examiner’s while he receives the following instruction: “do not hold my hands”).

***Emotional functioning assessment.*** In order to assess the presence of symptomatology, the Hospital Anxiety and Depression scale (HADS; Zigmond and Snaith, 1983), a self-report questionnaire, was employed. The HADS has demonstrated to be a sensitive tool to assess depression and anxiety symptoms in acquired brain injury population (Dawkins et al., 2006). To assess the use of reappraisal in daily life, the *Emotion Regulation Questionnaire* (Gross and John, 2003) was administered.

#### Reappraisal generation task

This task is adapted from several studies on reappraisal ability, and is described in detail in Salas et al. (2013). 10 pictures^1^ were selected from the International Affective Picture System (*IAPS*; Lang et al., 2008), depicting negative events of different sorts. These pictures were selected to cover the wide range of possible negative scenarios that people commonly face (death, natural disasters, accidents, illness, violence, etc.). The pictures were displayed in a 14′ computer screen.

At the beginning of the task participants were trained to generate reappraisals, using three practice IAPS pictures (not part of the set of 10 test pictures). The task was introduced as follows: “Sometimes people try to feel better by looking on the bright side of things. You will watch pictures of negative events and will be asked to think aloud about the positive side of these situations. Try to be *fast* and say *as many* positive sides you can think of.”

In order to avoid memory bias for the neurological group, on the computer screen, above each picture, the task instruction was summarized: “Think aloud about the positive side of this situation. Try to be quick.” Participants were informed that their answers would be timed, and recorded verbatim. They were also informed that the aim of the task was to produce as many positive reinterpretations as possible. If they were not able to generate a correct reappraisal for the first picture, several reappraisal examples were offered [e.g., Car Crash (9903): “when looking at this picture some people say that help is on the way” or “is not as bad as it looks”]. The same procedure was followed with the second and third practice pictures. Both the neurological group and the non-brain injured group were able to offer adequate reappraisals by the end of the three-picture practice phase.

## DATA ANALYSIS

In order to test *Hypothesis 1* (that participants with LH lesions will present a more marked impairment generating reappraisals compared to subjects with RH lesions and controls), two variables were generated, following a similar analysis to the one used by Salas et al. (2013). *Reappraisal difficulty* was obtained averaging the number of seconds that each subject required to offer the first reappraisal in each picture. *Reappraisal productivity *was measured by averaging the number of spontaneous reappraisal (with no prompting) generated during each picture. The two variables were compared between groups. Because normality and independence of variance assumptions were not met, a non-parametric test for differences between 3 or more groups (Kruskal–Wallis) was used. If the overall model showed significant differences, two planned comparisons were tested using Mann-Whitney test [healthy controls vs. brain injury patients (left + right hemisphere); LH vs. RH].

*Hypothesis 2* (that cognitive control abilities would be negatively associated to reappraisal difficulty, and positively related to reappraisal productivity) was addressed using correlational methods. As a first step, bivariate correlations were used to explore independent associations between reappraisal difficulty and reappraisal productivity with cognitive control abilities. As a second step, a multiple linear regression model was tested. From the seven initial tasks considered to assess the four cognitive processes, three were dropped. Two of them, related to inhibition (sensitivity to interference and environmental autonomy), did not show enough variability (most of the participants performed with the highest score). The two fluidity tasks (letter fluency and category fluency) showed a medium correlation between them (*r* = 0.5, *p* = 0.01). In consequence, category fluidity was dropped in order to decrease the number of parameters and avoid possible multincollinearity. The decision to preserve letter fluency was based on evidence suggesting that, compared to category fluency, it is more strongly associated to cognitive control abilities (Henry and Crawford, 2004).

## RESULTS

### COGNITIVE AND EMOTIONAL FUNCTIONING

A detailed description of the average scores of each group on the cognitive and emotional assessment can be found in **Table 2**. In relation to emotional functioning, it is interesting that all three groups had subclinical levels of anxiety (LH: *M* = 3.67; RH: *M* = 5; HC: *M* = 4.64) and depression (LH: *M* = 4.33; RH: *M* = 4.75; HC: *M* = 2.71). Nevertheless, compared to healthy controls, individuals with brain injury presented significantly higher scores in the depression scale (*p* = 0.039). In addition, none of the neurological groups differed from healthy controls in the self-reported use of reappraisal [*F*(2,22) = 2.38, *p* = 0.11].

**Table 2:** Cognitive and emotional performance of neurological groups and healthy controls.

	Task	LH Group		RH Group		HC Group		HC vs. BI	LH vs. RH
		***M***	***SD***	***M***	***SD***	***M***	***SD***
Overall cognition	Minimental state examination	25.67	2.88	27.83	1.60	29.17	1.34	*p *= 0.001***	*p* = 0.066
	Sustained attention (TEA)	7.67	1.52	7.83	1.83	11.00	1.78	*p < *0.001***	*p* = 0.478
	Divided attention (TEA)	9.00	3.60	11.16	5.11	11.22	1.73	*p* = 0.083	*p *= 0.488
	Comprehensive language (token test)	26.00	9.53	31.17	1.17	31.33	0.78	*p* = 0.039*	*p* = 0.098
	Memory (WMS-R) coding	6.67	2.50	13.00	3.84	14.58	2.97	*p* = 0.033*	* p* = 0.131
	Memory (WMS-R) free recall	8.67	3.05	14.00	4.33	16.83	3.51	*p* = 0.14*	* p* = 0.171
	Memory (WMS-R) recognition	13.67	0.57	13.33	1.36	13.75	1.60	*p* = 0.19	* p* = 0.573
	Executive functions (FAB total)	13.33	4.04	15.50	0.23	17.08	0.99	*p* = 0.022*	* p* = 0.367
Cognitive control	Working memory (digits, WAIS)	10.00	4.58	9.17	2.56	10.67	2.31	*p* = 0.022*	* p* = 0.664
	Inhibition, sensitivity to interference	2.83	0.41	2.63	0.51	2.91	0.30	*p* = 0.067	* p* = 0.841
	Inhibition, inhibitory control	2.00	1.92	2.50	0.92	2.58	0.79	*p* = 0.205	* p* = 0.383
	Inhibition, environmental autonomy	3.00	0.00	2.83	0.40	3.00	0.00	*p* = 0.433	* p* = 0.461
	Verbal fluency (DKEF-S) letter	7.67	5.13	7.50	2.43	10.71	3.27	*p* = 0.006**	* p* = 0.910
	Verbal fluency (DKEF-S) category	4.33	0.57	8.00	2.09	10.73	2.19	*p* < 0.001***	* p* = 0.003**
	Abstraction (similarities, WAIS)	6.67	2.08	10.00	3.69	11.64	3.50	*p* = 0.013***	* p* = 0.423
Emotional functioning	Emotional symptoms (HADS) anxiety	3.33	0.57	6.17	4.04	4.27	3.52	*p* = 0.14	* p* = 0.431
	Emotional symptoms (HADS) depression	4.00	6.92	6.00	5.40	2.55	1.86	*p* = 0.039*	* p* = 0.305
	Emotion regulation (ERQ) reappraisal	23.67	14.50	33.00	4.19	32.00	5.07	*p* = 0.16	* p* = 0.091

Individuals with brain injury (BI) present significant differences on most of the cognitive measures when compared to healthy control (HC). The left hemisphere group (LH) only differs from the right hemisphere group (RH) on categorical verbal fluency. The two columns of the right present the level of significance of these differences. *p < 0.05, **p < 0.01, ***p < 0.001.

In relation to cognitive functioning, individuals with brain damage differed significantly from healthy controls in almost every measure, with the exception of divided attention (*p* = 0.083), memory recall (recognition; *p* = 0.19), inhibitory control (*p* = 0.20) and environmental autonomy (*p* = 0.43). Marginal differences (*p* = 0.067) were found on another measure of inhibition, sensitivity to interference. It is interesting to note that, when comparing the performance of both brain injured groups, no significant differences were found across all cognitive measures, with the exception of category fluidity (*p* = 0.003), where people with LH lesions obtained lower scores.

### LATERALITY HYPOTHESIS

#### Reappraisal difficulty

The average number of seconds taken to generate a first reappraisal did not differ between the LH and RH groups. However, significant differences were found between patients with brain injury in general (LH + RH), and HC. A Kruskal–Wallis non-parametric test was used to compare the time taken by each group. It was observed that the number of seconds was significantly different between groups [*H*(2) = 10.77, *p* = 0.002; *mean ranking*: LH = 21.71, RH = 18.25, HC = 9.79]. According to planned comparisons, it was found that this difference was only significant, and had a large effect size, between the HC group and the brain injury group (LH + RH; *U* = 32, *Z* = -3.19, *p* < 0.001, *r* = 0.59), but not significant, and with a negligible effect size, between LH and RH groups (*U* = 25, *Z* = -0.24, *p* = 0.43, *r* = -0.04). In conclusion, our findings do not support the *LH Reappraisal Hypothesis*, for patients with LH and RH damage were equally slowed in generating reappraisals.

#### Reappraisal productivity

**FIGURE 1:**
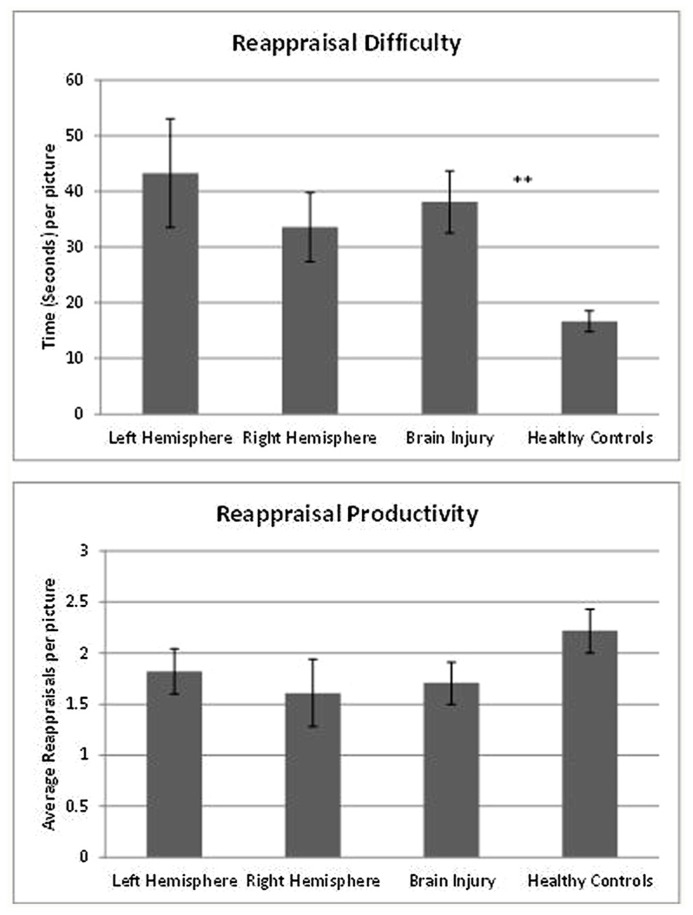
**Laterality and reappraisal generation.** The figures describe differences between neurological groups and controls in reappraisal *difficulty* and *productivity*. Patients with left hemisphere and right hemisphere lesions are equally slow generating the first reappraisal (top figure). However, they do not differ from controls in the average number of reappraisal produced in each picture (bottom figure), **p < 0.001.

The average number of reappraisals generated for each picture did not differ between groups, suggesting that individuals with LH and RH lesions, and healthy controls, are equally productive when time is not considered. A Kruskal–Wallis non-parametric test was used to compare the total reappraisals generated by each group. It was observed that the number of reappraisals was not significantly different between groups [*H*(2) = 3.51, *p* = 0.175; *mean ranking*: LH = 14.07, RH = 10.88, HC = 17.82]. A detailed description of the groups’ performance can be found in **Table 3**. A graphic summary of the results can also be found in **Figure 1**.

**Table 3:** Descriptive statistics of reappraisal generation and productivity, for both neurological groups and healthy controls.

	LH Group		RH Group		BI Group		HC	
	*M*	*SD*	*M*	*SD*	*M*	*SD*	*M*	*SD*
Reappraisal difficulty	43.3	25.88	33.58	17.6	38.13**	21.62	16.7	7.07
Reappraisal productivity	1.82	0.59	1.61	0.95	1.71	0.78	2.22	0.79

**p< 0.001.

### COGNITIVE CONTROL HYPOTHESIS

#### Reappraisal difficulty

From the four cognitive control processes considered, only two (inhibition and verbal fluency) were significantly associated with reappraisal difficulty. The final model tested had reappraisal difficulty as a dependent variable and working memory, abstraction, verbal fluency, and inhibition as predictors. Multicollinearity, homoscedasticity, and independency of errors assumptions were met. The model was significant [*F*(4,24) = 7.70, *p* < 0.001] and explained a 49% of the variance. The final model is presented in **Table 4**. From the four predictors, inhibition and verbal fluency were the only two significantly associated to reappraisal difficulty. By looking at the standard coefficient it is possible to conclude that Inhibition showed the strongest predictive value. In relation to the other two independent variables, abstraction was marginally related and working memory showed no relationship.

**Table 4:** Multiple linear regression model for reappraisal difficulty.

	Unstandardized coefficients		Standardized coefficients		
	*B*	Standard error	*B*	*t*	Significant
(Constant)	87.12	12.59		6.92	0.00
Working memory (digits)	0.26	0.93	0.04	0.28	0.78
Abstraction (similarities)	-1.64	0.87	-0.27	-1.88	0.07
Verbal fluency (letter)	-2.28	0.9	-0.37	-2.53	0.02*
Inhibition (inhibitory control)	-10.27	3.07	-0.47	-3.35	0.003*

The table describes the percentage of variance explained by each cognitive control process. Inhibition and verbal fluency are the only two significant variables. Abstraction presents a marginal relationship with reappraisal difficulty. *p < 0.05.

#### Reappraisal productivity

The same model was tested for reappraisal productivity. Contrary to the previous model, this one was not significant [adjusted *R*^2^ = 0.022, *F*(4,24) = 1.16, *p* = 0.35].

### RESULTS SUMMARY

Findings do not support the *Laterality Hypothesis*, challenging the assumption that LH lesions generate a more marked impairment in the generation of a first reappraisal (reappraisal difficulty) or the total number of reappraisal that can be produced (reappraisal productivity). Nevertheless, the results of this study suggest that having a brain injury in either hemisphere does have an impact on reappraisal difficulty. This pattern is not observed in relation to reappraisal productivity, where participants with brain injury reach similar levels than controls. In conclusion, participants with brain injury are slower, but equally productive than people with no brain injury when time constrains are not considered.

The results of this study appear to support the *Cognitive Control Hypothesis*, suggesting that the velocity to generate a first reappraisal, which is compromised in brain-injured patients in general, is associated with two cognitive control abilities. Thus, inhibition and verbal fluency present both negative relationships to reappraisal difficulty. Regarding abstraction, it only exhibits a marginally significant negative association. Working memory does not appear to be associated to the amount of time required to reappraise. Interestingly, no cognitive control variable appears to be associated to reappraisal productivity.

### DISCUSSION

In the past decade, there has been a growing interest in examining the neuroanatomical basis of reappraisal via neuroimaging. These studies have suggested that reappraisal tasks activate a set of areas in the LH that are commonly associated with language and cognitive control. The goal of this study was to answer two questions that flow from this observation. (1) Are participants with unilateral lesions to the LH more impaired than participants with RH lesions and neurologically healthy controls on a reappraisal generation task? (2) Which cognitive control abilities are associated with reappraisal generation?

#### Hemispheric laterality and reappraisal generation

Our focus in this study was reappraisal generation, or the capacity to *produce* positive reinterpretations of negative events. This is an important point to clarify, because most behavioral and neuroimaging studies to date have focused on reappraisal *ability*, or the capacity to modulate emotion through the use of reappraisal. Such emphasis on ability is sensible, considering that people with healthy brains probably have an intact capacity to *generate* reappraisals. However, a robust set of evidence on the neuropsychological consequences of brain injury suggests that the generation and manipulation of thoughts can be compromised by lesions to diverse brain areas (Goldstein and Scherer, 1941; Luria, 1966; McGrath, 1991; Gomez Beldarrain et al., 2005). If reappraisal relies on the flexible use of thinking (Ochsner and Gross, 2004), then it is likely that people with brain injury may struggle using such strategy to modulate how they feel.

Data from this study suggest that lesions to the LH, which has been long related to language functions (Frost et al., 1999; Corballis et al., 2012), and verbally mediated cognitive control (Stephan et al., 2003; Gruber and Goschke, 2004; Henry and Crawford, 2004), do not impair reappraisal generation more than lesions to the RH. This is interesting, since reappraisal has been mostly considered as a language- mediated ER strategy (Gross and Thompson, 2007; Mcrae et al., 2012a). A possible explanation for this negative finding may be related to the fact that LH and RH groups presented a similar profile of cognitive impairment (see **Table 2**). It is also possible that individuals with RH lesions presented low scores on reappraisal difficulty as a consequence of impairment of other non-language mechanisms, for instance, attention. According to our data, both groups are impaired on their ability to sustain attention, a deficit that is commonly expressed in everyday life as distractibility (Leclercq et al., 2002). If reappraisal involves the manipulation of attentional focus (Ochsner et al., 2002), it is possible that attentional deficits may have an impact on reappraisal generation. However, this explanation remains speculative and requires scientific exploration. 

An alternative explanation for this negative finding may well be related to the small sample size of the study. Even though there is a small difference between LH and RH groups, suggesting that people with LH lesions are slower than RH individuals generating reappraisals, this does not appear significant. In addition, this negative finding may also be interpreted in view of the unbalanced distribution -inside each neurological group- between anterior and posterior lesions. In fact, the LH group presented less individuals with prefrontal damage (anterior = 3, posterior = 5) compared to the RH group (anterior = 7, posterior = 1), perhaps compromising comprehensive (posterior), and not executive (anterior) aspects of language. In the future, new studies should consider this variable and generate experimental designs that include individuals with lesions to anterior and posterior areas of each hemisphere. Considering the existing neuroimaging literature on reappraisal (see Introduction), it is possible that damage to the anterior portions of the LH will have a more marked impact on the capacity to generate *reappraisals* than damage to posterior areas of the left convexity.

#### Neuropsychological components of reappraisal generation

The available literature on cognitive control and reappraisal is scarce and focuses exclusively on reappraisal ability. So far, it has been suggested that reappraisal ability is positively related to working memory (Schmeichel et al., 2008; Mcrae et al., 2012b) and marginally related to abstraction (Mcrae et al., 2012b). In relation to verbal ability (e.g., to *generate* alternative interpretations), only one study has found indirect evidence that verbal fluency is related to reappraisal ability, however, this on a suppression task (Gyurak et al., 2009). Interestingly, no associations have been reported for inhibition, a key theoretical aspects of reappraisal (e.g., to *detach* from the negative emotional experience).

This study suggests that an inhibitory control task (Drewe, 1975), where participants must withhold a response that was given to the same stimulus before (e.g., tap once when the examiner taps twice), can significantly predict the amount of time that it takes to generate a first reappraisal. In other words, if an individual decreases in one point his score in the inhibition task (Dubois et al., 2000), his response time to generate a first reappraisal will increase by 10.27 s. This finding is the first to support the view that inhibition is a key ability in decreasing the salience of automatic negative appraisals (Mcrae et al., 2012b), for when inhibition is impaired, reappraisal generation requires considerably more time. There is one case study that has reported this relationship in detail (Salas et al., 2013), describing how inhibition impairment after a left fronto-parietal lesion produced a remarkable difficulty to spontaneously generate reappraisals. In addition, this finding is also consistent with a large literature associating damage to the left and right PFC with deficits withholding prepotent responses (Aron et al., 2003, 2004; Swick et al., 2008). Finally, it is important to note that this data is also consistent with models of executive function that propose behavioral inhibition as a requisite to any goal directed behavior (Barkley, 1997, 2001).

The results obtained in this experiment also suggest that performance on a verbal fluency task (Delis et al., 2001), where participants are expected to generate as many words that begin with a letter in one minute, also predict the amount of time taken to generate a first reappraisal (albeit to a less extent than inhibition). Verbal fluency is an interesting measure to consider in the context of reappraisal, both because deficits in verbal fluency are a common feature of prefrontal cortex damage (Henry and Crawford, 2004; Robinson et al., 2012), and also because verbal fluency presents high associations with verbal ability (Miller, 1984; Crawford et al., 1992, 1993; Sollberger et al., 2010), a core component of reappraisal (Mcrae et al., 2012b). In addition, verbal fluency also appears to recruit other processes that are key to reappraisal, such as the retrieval and recall of information, self- monitoring and inhibition (Perret, 1974; Henry and Crawford, 2004).

**FIGURE 2:**
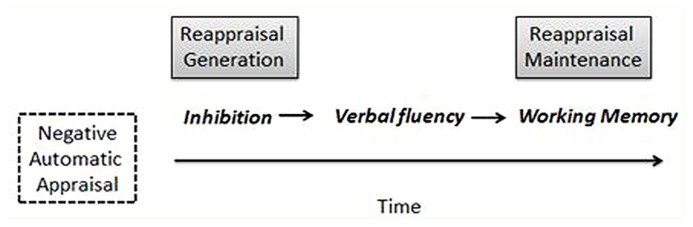
**A two-stage process of reappraisal.** On a first stage (*reappraisal generation*) automatic negative meanings are inhibited and new positive, or less negative, meanings are generated. On the second stage (*reappraisal maintenance*) positive, or less negative, new meanings are kept in mind and protected from competing negative appraisals. Inhibition and verbal fluency are key abilities for reappraisal generation, while working memory is central for reappraisal maintenance.

Based on these findings, and considering data from previous studies on reappraisal ability, a two-stage reappraisal model may be proposed (see **Figure 2**). In a first stage (*reappraisal generation*) inhibition is required to disengage from the automatic negative meaning. If inhibition is successful, alternative interpretations, or new meanings, can be generated -this moderated by verbal fluency. In addition, and considering previous data (Schmeichel et al., 2008; Mcrae et al., 2012b), it is also suggested that, during a second phase (*reappraisal maintenance*), working memory ability has a role keeping in mind the recently generated new interpretation, thus “shielding” it from the initial meaning that still remains in the focus of attention (Kanske et al., 2010; Gross, 2013). This model is consistent with the implementation-maintenance model of reappraisal, IMMO (Kalisch, 2009), which suggests that the process of reappraisal has early and late components. According to this model, early components refer to operations needed for *choosing* and *implementing* an initial reappraisal strategy, while late components are required to *maintain* a reappraisal in working memory and *monitor* its success. Taken together, data from this lesion study suggests that inhibition and verbal fluency, two neuropsychological abilities, are critical in implementing an initial reappraisal strategy during the early phase of the process. Working memory, on the contrary, seems not to be relevant in generating (implementing) a reappraisal during the early moments of the process.

#### Reappraisal generation impairment and neuropsychological rehabilitation

This study offers insights into how brain damage may compromise ER. It suggests that brain injury, without regard to lesion laterality, impairs the capacity to quickly generate positive reinterpretations of negative events. However, when longer periods of time are provided, patients are able to produce reappraisals as well as controls. These findings are relevant to neuropsychological rehabilitation for two reasons.

First, they offer supporting evidence to the idea that brain-injured patients in general are vulnerable to experience emotion dysregulation (Salas, 2012), particularly when confronted to emotional situations where they need to react quickly. This is consistent with studies describing that focal and non-focal brain damage compromises the capacity to rapidly react to environmental demands (Goldstein, 1995; Gerritsen et al., 2003; Winkens et al., 2006; Mathias and Wheaton, 2007), and that negative emotional events can compromise cognitive abilities (e.g., Demanet et al., 2011).

Second, they suggest that a psychological capacity such as reappraisal can be externally modulated, this, by manipulating environmental demands (time). In other words, people with acquired brain injury are slower than controls, but if enough time is offered, they are equally able to generate reappraisals, at least in the case of relatively high-functioning individuals such as those studied in the present research. This is in line with evidence proposing that neuropsychological impairments are not stable, but can be modulated by physical or interpersonal context (Bowen et al., 2010, for a review). For example, it has been reported that the use of prompts facilitates the process of disengagement from negative visual material, increasing dramatically the capacity to generate positive reinterpretations (Salas et al., 2013). This evidence appears to support the theoretical proposition that extrinsic forms of ER, such as affective and cognitive engagement (Niven et al., 2009), can be used to compensate for intrinsic ER difficulties (reappraisal; Freed, 2002; Salas, 2012).

## CONCLUSION

In recent years, scientists have become increasingly interested in the neural bases, and neuropsychological foundations, of ER. This study contributes to the literature by exploring the role of each hemisphere, and the relevance of several cognitive control abilities, in reappraisal generation. In addition, and perhaps most importantly, this study has addressed these issues using a well-known ER paradigm on a sample of individuals with acquired brain injury. Such an approach opens important possibilities to ER research, complementing behavioral and neuroimaging studies with non-clinical participants.

## Conflict of Interest Statement

The authors declare that the research was conducted in the absence of any commercial or financial relationships that could be construed as a potential conflict of interest.

## Footnotes

1 Pictures’ theme and IAPS number: Riot (2691), Shoplifting (2745), Sick baby (3350), Burnt building (9471), Funeral (2799), Tornado (5971), Pollution (9341), Flood (9926), Car crash (9903), Graveyard (9220). The picture set had an overall negative valence (*M* = 3.01; *SD* = 0.8) according to the IAPS database.
